# Disposal of the Dead

**Published:** 1879-04

**Authors:** 


### DISPOSAL OF THE DEAD.

We are pre-eminently creatures of habit; this is daily demonstrated in all our pursuits; we are loth to leave well trodden paths to the neglect of broader avenues. We find verification of the fact in our dislike for any innovation in the method of transacting business or labor. Years were required, before we could be prevailed upon to use the various labor-saving machines, that have since become invaluable to us. The farmer preferred the scythe and sickle in reaping his harvest, rather than save time and muscle by using the now universally employed reapers and mowers. The house wife sat and sewed late into the night, rather than shorten the hours by using the sewing machine that is now considered indispensable in every household. Habit and usage is still more strictly adhered to in our religious ceremonies. Why we should retain the customs of the ancients, does not clearly appear; unless it be thought sacriligeous to make any innovations upon the ways of our ancestors. The method of disposing of our dead is as susceptible of, and clearly needs improvment, and will be as great a benefit to the people, as any of the changes first noted. It has been shown that the present custom of burying the dead, induces disease and suffering among the living by poisoning the water sources; does it not seem irrational if not absolutely criminal to persist longer in it ?

Dr. Gregg, whose ability as an analytical chemist is widely known, certifies us that wells in the neighborhood of cemeteries are poisoned by decomposing animal matter, the virulence of which is not destroyed even by percolation through the ground to remote distances.

Examination of the water from these wells under the microscope, also reveals living infusoria that find lodgment in the human body, giving rise to various diseases. Does not this condition of affairs call loudly for a remedy ? Every rational mind must answer affirmatively. This is offered us then, in cremation. No cheaper, cleanlier, more rational way, can be devised; no argument can be offered against it that will stand, and nothing appears opposed to it save the simple matter of *custom* to which we have tenaciously adhered for centuries.

When cremation becomes the custom, as some day it will, we will all wonder why the filthy burial system was so long persisted in.

